# Leveraging Large Language Models for Predicting Postoperative Acute Kidney Injury in Elderly Patients

**DOI:** 10.34133/bmef.0111

**Published:** 2025-03-11

**Authors:** Hanfei Zhu, Ruojiang Wang, Jiajie Qian, Yuhao Wu, Zhuqing Jin, Xishen Shan, Fuhai Ji, Zixuan Yuan, Tingrui Pan

**Affiliations:** ^1^ Center for Intelligent Medical Equipment and Devices, Institute for innovative Medical Devices, School of Biomedical Engineering, Division of Life Sciences and Medicine, University of Science and Technology of China, Hefei, Anhui, P. R. China.; ^2^ Suzhou Institute for Advanced Research, University of Science and Technology of China, Suzhou, Jiangsu, P. R. China.; ^3^ Department of Anesthesiology, The First Affiliated Hospital of Soochow University, Suzhou, Jiangsu, P. R. China.; ^4^ Department of Nephrology, The Second Affiliated Hospital of Anhui Medical University, Hefei, P. R. China.; ^5^ Thrust of Financial Technology, The Hong Kong University of Science and Technology (Guangzhou), Guangzhou, Guangdong, P. R. China.; ^6^ Department of Precision Machinery and Precision Instrumentation, School of Engineering Science, University of Science and Technology of China, Hefei, Anhui, P. R. China.

## Abstract

**Objective:** The objective of this work is to develop a framework based on large language models (LLMs) to predict postoperative acute kidney injury (AKI) outcomes in elderly patients. **Impact Statement:** Our study demonstrates that LLMs have the potential to address the issues of poor generalization and weak interpretability commonly encountered in disease prediction using traditional machine learning (ML) models. **Introduction:** AKI is a severe postoperative complication, especially in elderly patients with declining renal function. Current AKI prediction models rely on ML, but their lack of interpretability and generalizability limits clinical use. LLMs, with extensive pretraining and text generation capabilities, offer a new solution. **Methods:** We applied prompt engineering and knowledge distillation based on instruction fine-tuning to optimize LLMs for AKI prediction. The framework was tested on 2,649 samples from 2 private Chinese hospitals and one public South Korean dataset, which were divided into internal and external datasets. **Results:** The LLM framework showed robust external performance, with accuracy rates: commercial LLMs (internal: 63.73%, external: 68.73%), open-source LLMs (internal: 63.70%, external: 64.24%), and ML models (internal: 63.93%, external: 58.27%). LLMs also provided human-readable explanations for better clinical understanding. **Conclusion:** The proposed framework showcases the potential of LLMs to enhance generalization and interpretability in postoperative AKI prediction, paving the way for more robust and transparent predictive solutions in clinical settings.

## Introduction

Acute kidney injury (AKI) refers to a rapid decline in renal function, characterized by a marked reduction in glomerular filtration rate, which leads to an inability to effectively remove waste and fluids from the body, resulting in complications and, in severe cases, death [1]. Postoperative AKI constitutes a substantial proportion of all AKI cases, with an incidence rate as high as 57.1% [2,3]. Elderly patients are particularly susceptible to postoperative AKI due to the natural decline in renal function with age and the frequent presence of chronic conditions such as hypertension and diabetes, which increase their sensitivity to intraoperative medications and reduce their tolerance to hemodynamic fluctuations and postoperative complications [4]. Recent study has showed that the incidence of postoperative AKI is significantly higher in elderly patients compared to younger patients [5]. Therefore, postoperative AKI not only is a significant contributor to cases of AKI but also poses a higher risk for elderly patients. Given this, early prediction of postoperative AKI in elderly patients, followed by timely intervention, is crucial [1,6].

Currently, AKI prediction models typically leverage machine learning (ML) models [7], but they face 2 significant challenges in clinical application. First, many ML models are “black-box” algorithms with poor interpretability [8], making it difficult for clinicians to understand the specific reasons behind the model’s predictions. This lack of transparency limits the model’s trust and adoption in clinical settings, especially for elderly patients, whose physiological conditions are more complex and vary significantly [9,10]. In such cases, doctors must understand the rationale behind the model’s decisions to provide personalized treatment plans. Second, ML models are often trained on internal datasets for parameter updates, which can lead to overfitting when applied to external datasets [11,12]. Due to differences in feature distributions between datasets, the model’s predictive performance on external data typically declines, with studies showing a drop of 7% to 20% [13–15]. The higher heterogeneity of characteristics in elderly populations further constrains the model’s generalizability and limits its effectiveness across diverse clinical settings.

In recent years, the rapid development of large language models (LLMs) has brought revolutionary changes to various fields, including healthcare [16]. Previous studies have explored the application of LLMs in processing electronic medical records and analyzing medical texts [17]. However, the potential of LLMs in disease prediction, particularly for complex conditions like AKI, remains underexplored. LLMs possess powerful reasoning and natural language processing capabilities [18], which could address the challenges of poor interpretability and limited generalization in AKI prediction models. First, LLMs can generate easily understandable natural language explanations, providing clinicians with clear rationales behind predictions, significantly enhancing model interpretability, and resolving the transparency issues inherent in traditional black-box models. Second, as LLMs are pretrained on large-scale, diverse datasets [19], they are better equipped to adapt to the data distributions of different hospitals and patient populations, improving the model’s external generalization ability. This is particularly important for predicting postoperative AKI in elderly patients, who exhibit greater diversity in clinical characteristics and require models with stronger adaptability. These features suggest that LLMs have the potential to be a key tool in improving AKI prediction models, offering more reliable support for the early identification of high-risk patients and ultimately enhancing patient outcomes.

Here, we propose a predictive framework for predicting the risk of postoperative AKI in elderly surgical patients and evaluate their practicality in real clinical settings. Through prompt engineering and knowledge distillation [20], we enhanced the capabilities of both closed-source commercial and open-source LLMs to predict postoperative AKI outcomes and generate detailed explanations based on preoperative and intraoperative data from elderly patients. These models respectively provide more accurate predictions and stronger privacy protection. We developed and tested these models using real clinical data from elderly surgical patients at the First Affiliated Hospital of Soochow University and further validated them externally with clinical cases from Henan Provincial People’s Hospital and a public database from Korea. The results show that LLMs successfully generated human-readable text explaining and achieved performance comparable to commonly used ML models on internal datasets (best accuracy in Suzhou: commercial LLMs: 63.73%; open-source LLMs: 63.70%; ML models: 63.93%) while significantly outperforming ML models on external datasets (best accuracy in Henan: commercial LLMs: 68.73%; open-source LLMs: 64.24%; ML models: 58.27%), demonstrating the models’ strong generalization and versatility. This study highlights the potential of LLMs in clinical disease reasoning and prediction, offering new insights and directions for their application in disease prediction.

## Results

### Overview of preoperative AKI prediction framework

**Fig. 1.**
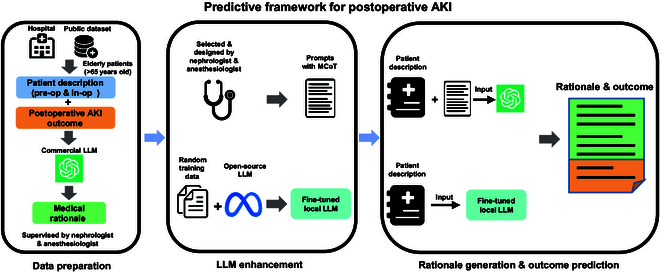
An overview of postoperative AKI prediction framework. Under this framework, we utilize preoperative and intraoperative patient data to predict postoperative AKI outcome and provide a rationale for the predictions. Initially, medical rationale examples are generated using data of patients’ description and their postoperative AKI outcomes under physician supervision. These examples are employed to create MCoT dataset used by commercial LLMs, as well as instruction fine-tuning data for open-source LLMs to enhance models’ capabilities. The patients whose data were not used for prompting and fine-tuning are reserved for testing, with the patient descriptions input into both the commercial LLMs and locally fine-tuned LLMs to generate corresponding medical rationales and predicted postoperative outcomes.

Figure 1 presents the overall framework for our AKI prediction approach in elderly surgical patients. Within this framework, we analyze preoperative and intraoperative information to assess postoperative AKI risk factors and predict patient outcomes.

Under the supervision of physicians, we input descriptions of patients along with their postoperative outcomes into a commercial LLM. The LLM is instructed to engage in step-by-step reasoning, providing medical principles that lead to these outcomes. By combining the generated medical principles with the patients’ descriptions and outcomes, we derive a set of Medical Chain of Thought (MCoT) prompts. This dataset is subsequently used for prompt engineering and knowledge distillation.

To enhance the capabilities of closed-source commercial LLMs for outcome prediction and medical rationale generation, 10 representative cases have been selected from the MCoT dataset by the physician, which will serve as candidate prompts for further use. For open-source LLMs, we randomly selected training samples from the MCoT dataset for the knowledge distillation process. Instruction fine-tuning is used to distill knowledge from the commercial LLMs into local, low-parameter open-source LLMs. Specific parameters in the local LLMs are adjusted through instruction fine-tuning, enabling the model to more effectively predict postoperative AKI outcomes in elderly patients based on the training data.

To generate predictive explanations and outcomes, we combined patient descriptions with candidate MCoT prompts and input them into commercial LLMs, enabling a step-by-step analysis of postoperative AKI risk factors. This approach helps commercial LLMs better understand patient conditions, resulting in more accurate predictive explanations and postoperative outcomes. For open-source LLMs, fine-tuned local models can output relevant medical principles and AKI outcome predictions based on patient descriptions, following the instructions used during training.

### Cohort characteristics

This study utilized 3 datasets from different medical institutions, comprising a total of 2,649 patients. Among them, 1,544 patient records were extracted from the electronic health records (EHRs) of The First Affiliated Hospital of Soochow University (hereafter referred to as Suzhou), 1,004 records were obtained from the EHR of Henan Provincial People’s Hospital (hereafter referred to as Henan), and 101 records were sourced from the public VitalDB [21] dataset. Of all patients, 1,323 (49.43%) experienced postoperative AKI. All patients were over the age of 65 and underwent various surgeries, including cardiac, general, thoracic, gynecological, orthopedic, and urological surgeries. The anesthesia types involved were general anesthesia and regional anesthesia.

The internal datasets included detailed patient information with 86 features (Table 1), including 2 demographic characteristics, 9 preoperative comorbidities, 2 preoperative medication characteristics, 23 preoperative laboratory test results, and 49 surgery and anesthesia-related characteristics (e.g., surgery duration, anesthesia duration, ASA (American Society of Anesthesiologists) score, intraoperative monitoring, and intraoperative medications), as well as postoperative AKI outcomes. Detailed patient information can be found in Table S1. Compared to the Suzhou and Henan datasets, the VitalDB dataset lacked certain preoperative comorbidity information, laboratory test results, and intraoperative medication features. To ensure the proper functioning of specific ML models, we describe how we handled these missing values in the Methods section. To prevent the impact of imbalanced data on model evaluation, the ratio of patients with postoperative AKI to those without AKI in the Suzhou, Henan, and VitalDB datasets was approximately 1:1.

**Table 1.** Part of study clinical characteristics of the Suzhou, Henan, and VitalDB datasets

Dataset	Suzhou (internal)	Henan (external)	VitalDB (external)
Number of patients (number of postoperative AKI patients)	1,544 (772)	1,004 (500)	101 (51)
Gender (M:F)	869:675	555:449	72:29
Age (mean, SD)	71.76 (5.92)	73.16 (6.79)	71.93 (5.54)
Number of preoperative characteristics	38	38	19
Hypertension (number, %)	725 (46.96)	248 (24.73)	48 (47.52)
Diabetes (number, %)	210 (13.60)	213 (21.22)	15 (14.85)
Glucose, mM (mean, SD)	5.84 (1.97)	5.95 (2.28)	7.30 (2.92)
Blood urea nitrogen, mM (mean, SD)	6.07 (2.60)	6.49 (2.45)	1.08 (0.78)
Creatinine, μM (mean, SD)	70.25 (39.81)	70.24 (32.21)	101.18 (117.98)
……			
Number of interoperative characteristics	45	45	15
General anesthesia (number, %)	1,431 (92.68)	792 (78.88)	99 (98.02)
Duration of anesthesia, min (mean, SD)	168.48 (121.63)	178.46 (120.94)	261.64 (139.23)
Intraoperative blood loss, ml (mean, SD)	178.60 (377.15)	172.60 (318.23)	919.98 (2,618.24)
Duration of interoperative hypotension, min (mean, SD)	13.90 (31.73)	54.21 (77.98)	17.23 (34.57)
Use of deoxyepinephrine (number, %)	152 (9.84)	2 (0.20)	35 (34.65)
……			

### Performance of commercial LLMs under prompt engineering

First, we evaluated the performance of commercial LLMs in predicting postoperative AKI outcomes in elderly patients using prompt engineering (Table 2). Specifically, we selected the GPT-4o and GPT-4o mini models for subsequent testing and employed both standard example shots and MCoT methods to enhance model performance. When using standard examples, we tested performance under zero-shot and 2-shot conditions. In the zero-shot setting, we directly input patient descriptions and asked the model to predict postoperative AKI outcomes. In the 2-shot setting, we first provided 2 patient descriptions along with their postoperative outcomes and then asked the model to predict the AKI outcomes for a new patient. In most cases, the model’s precision was higher in the 2-shot scenario than in zero-shot, while recall was lower. This indicates that after being provided with example information, the LLMs improved their accuracy in predicting positive AKI cases. However, without example prompts, the model tended to make more aggressive predictions, anticipating that more patients would develop postoperative AKI, resulting in a higher recall.

**Table 2.** Performance (%) of GPT-4o and GPT-4o mini on the Suzhou, Henan, and VitalDB datasets under zero-shot, few-shot, zero-shot MCoT, and few-shot MCoT (bolded numbers represent top performance)

		Suzhou (internal)			Henan (external)			VitalDB (external)		
Model	Prompt	Accuracy	Precision	Recall	Accuracy	Precision	Recall	Accuracy	Precision	Recall
GPT-4o	0-shot	57.78	59.08	70.34	59.36	59.71	75.00	50.50	53.66	86.27
	2-shot	58.29	61.19	45.34	63.65	63.58	63.20	65.35	62.90	76.47
	0-shot MCoT	60.62	58.38	**74.48**	63.05	59.37	**82.40**	58.42	55.17	**94.12**
	2-shot MCoT	62.50	**65.24**	53.50	67.93	**68.38**	66.60	68.31	64.62	82.35
	4-shot MCoT	62.88	64.13	59.97	68.23	65.12	78.40	67.33	64.61	82.35
	10-shot MCoT	**63.73**	63.11	66.71	**68.73**	65.82	78.20	**70.30**	**65.67**	86.27
GPT-4o mini	0-shot	58.35	55.74	**81.09**	58.17	55.24	84.40	59.41	55.68	**96.08**
	2-shot	59.97	58.37	69.56	59.86	57.38	75.40	57.42	54.44	**96.08**
	0-shot MCoT	55.05	53.56	76.04	61.45	57.23	**89.40**	58.42	55.17	94.11
	2-shot MCoT	59.52	62.23	48.45	65.74	**68.40**	58.00	62.38	**66.27**	64.71
	4-shot MCoT	61.20	58.86	74.87	65.74	62.19	79.60	64.36	60.00	88.24
	10-shot MCoT	**61.46**	58.97	75.78	**66.04**	61.88	82.80	**65.35**	60.26	92.16

Studies have shown that the Chain of Thought (CoT) prompting approach—encouraging the model to think step-by-step before answering—significantly enhances LLM performance, especially for complex mathematical problems [22]. We tested whether this approach could improve AKI outcome prediction and, to this end, designed an MCoT prompting method. This approach prompted the model to gradually analyze the risk factors for postoperative AKI before making a final prediction. We tested the model’s performance under zero-shot MCoT and various few-shot MCoT scenarios. With MCoT, GPT-4o’s performance on the Suzhou dataset improved significantly, reaching 60.62% in the zero-shot MCoT setting and achieving optimal performance of 63.73% with 10-shot MCoT. Notably, even when MCoT examples were derived from the Suzhou dataset, the approach still improved the model’s performance on external datasets. For instance, with 10-shot MCoT, the prediction accuracy for the Henan dataset increased from 59.36% to 68.73%, and for the VitalDB dataset, it rose from 50.50% to 70.30%. This demonstrates that MCoT plays a crucial role in activating the predictive capacity of commercial LLMs. Once this capacity is activated, the model’s ability to analyze patient information step-by-step leads to better predictions on both internal and external datasets, indicating effective generalization under the MCoT prompting method.

Overall, GPT-4o outperformed GPT-4o mini. As a lower-parameter version designed for quicker responses in smaller tasks, GPT-4o mini may be less effective in handling complex problems, such as postoperative AKI prediction, which require step-by-step reasoning.

### Performance of open-source LLMs after knowledge distillation

Considering the privacy concerns associated with medical data and the potential need for future model customization, we utilized 2 open-source models: Qwen2-7B-Instruct (hereafter referred to as Qwen2) and Llama3.1-8B-Instruct (hereafter referred to as Llama3.1). We chose Qwen2 and Llama3.1 as they were recent open-source LLMs that demonstrated strong performance at the time our experiments were conducted. We distilled knowledge from commercial LLMs to these open-source models through instruction fine-tuning, establishing locally deployable LLMs for postoperative AKI prediction. We also validated the performance of the open-source models before and after fine-tuning on various datasets (Table 3). The fine-tuning data came from the Suzhou dataset, consisting of 544 cases used for fine-tuning and the remaining 1,000 cases for testing.

**Table 3.** Performance (%) of Qwen2 and Llama3.1 on the Suzhou, Henan, and VitalDB datasets before and after instruction fine-tuning.(Qwen2 represents Qwen2-7B-Instruct, Llama3.1 represents Llama3.1-8B-Instruct)

		Suzhou (internal)			Henan (external)			VitalDB (external)		
Model		Accuracy	Precision	Recall	Accuracy	Precision	Recall	Accuracy	Precision	Recall
Qwen2	Base	49.90	51.00	99.20	52.09	52.23	89.00	50.50	51.07	92.16
	Fine-tuning	61.30	67.05	47.20	62.25	64.89	53.60	58.42	60.71	66.67
Llama3.1	Base	54.30	52.82	82.40	54.38	52.64	85.60	57.43	54.88	88.24
	Fine-tuning	63.70	65.47	58.40	64.24	62.48	71.60	60.39	57.97	78.43

The results showed that, without instruction fine-tuning, both Qwen2 and Llama3.1 performed poorly in predicting postoperative AKI, with prediction accuracies of 49.90% and 54.30% on the Suzhou dataset, respectively, which are close to random guessing. After instruction fine-tuning, their performance improved significantly, with accuracies rising to 61.30% and 63.70%, respectively. This indicates that the fine-tuned models are better equipped to understand and process complex medical information.

Moreover, the fine-tuned LLMs demonstrated considerable improvement in prediction accuracy on external datasets and displayed stable performance. This suggests that instruction fine-tuning enhances the models’ ability to predict based on data from a single medical institution and shifts their capabilities toward the specific task of AKI prediction. Consequently, the models achieved better generalization for postoperative AKI prediction.

**Fig. 2.**
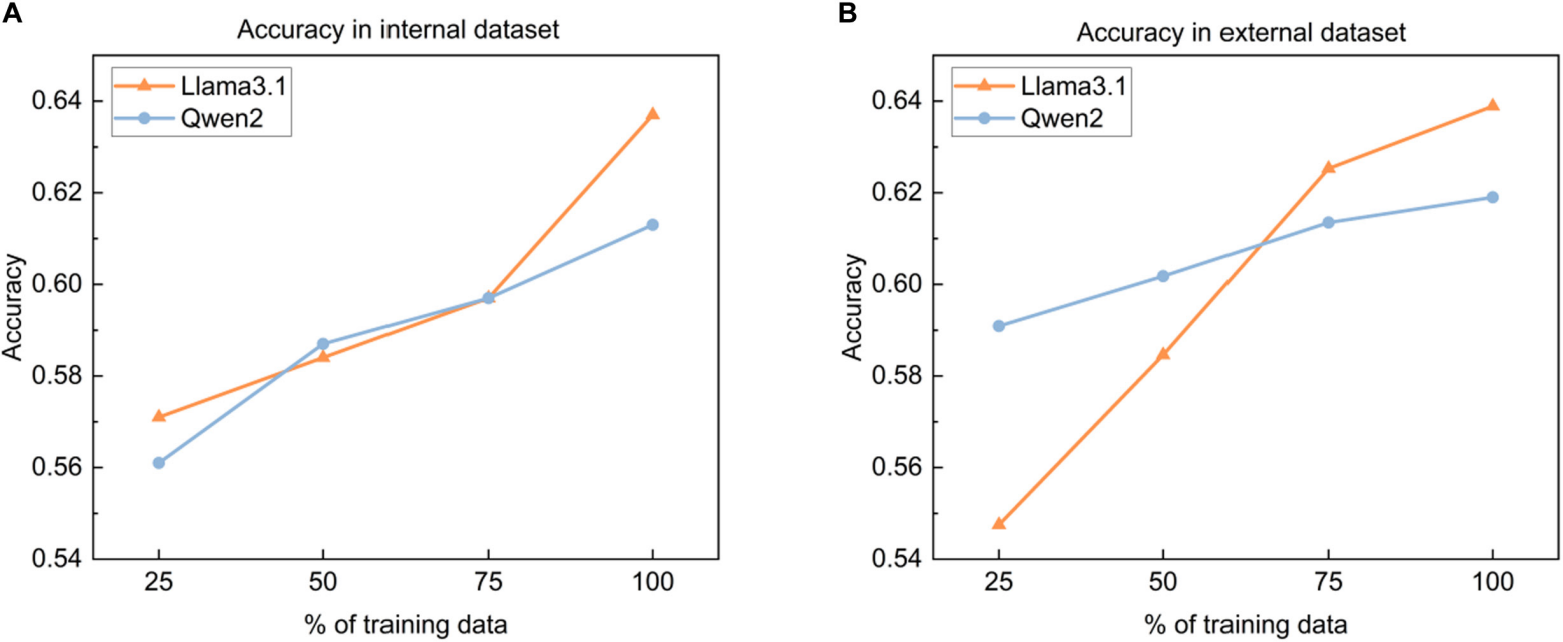
The impact of fine-tuning data volume on prediction performance. The predictive performance of Qwen2 and Llama3.1, after fine-tuning on 25%, 50%, 75%, and 100% of the training data, was evaluated. In the figures, the light blue line represents the prediction accuracy of Qwen2, while the orange line represents the prediction accuracy of Llama3.1. (A) Performance of the open-source LLMs on the Suzhou internal dataset across different proportions of fine-tuning data. (B) Performance of the open-source LLMs on external datasets, which include data from Henan and VitalDB, across different proportions of fine-tuning data.

To investigate whether the volume of fine-tuning data affects the predictive capabilities of local models, we evaluated their performance with varying amounts of fine-tuning data (Fig. 2). The results show a clear upward trend in prediction accuracy for postoperative AKI as the fine-tuning data volume increases for both Qwen2 and Llama3.1. This indicates that learning from more examples of postoperative AKI enhances the predictive capabilities of low-parameter local LLM models.

We observed that Qwen2’s most significant accuracy improvement on the internal dataset occurred when the fine-tuning data volume increased from 25% to 50%. For Llama3.1, the largest accuracy gain was seen when the fine-tuning data volume increased from 75% to 100%. This suggests that, due to differences in the underlying model architectures, the impact of fine-tuning data volume on model performance varies.

### Comparison of LLMs and traditional ML prediction models

Many studies on AKI prediction utilize ML models [12,23]. These models continuously adjust their parameters using the training dataset to minimize errors between predicted and actual outcomes, resulting in more accurate predictions. In this study, we selected the best-performing models from previous AKI prediction research [23–26], including the ensemble model XGBoost, Random Forest, the tree-based model Decision Tree, and the linear model Logistic Regression. These models were trained and tested on the Suzhou dataset and subsequently used to generate predictions for the Henan and VitalDB datasets.

**Fig. 3.**
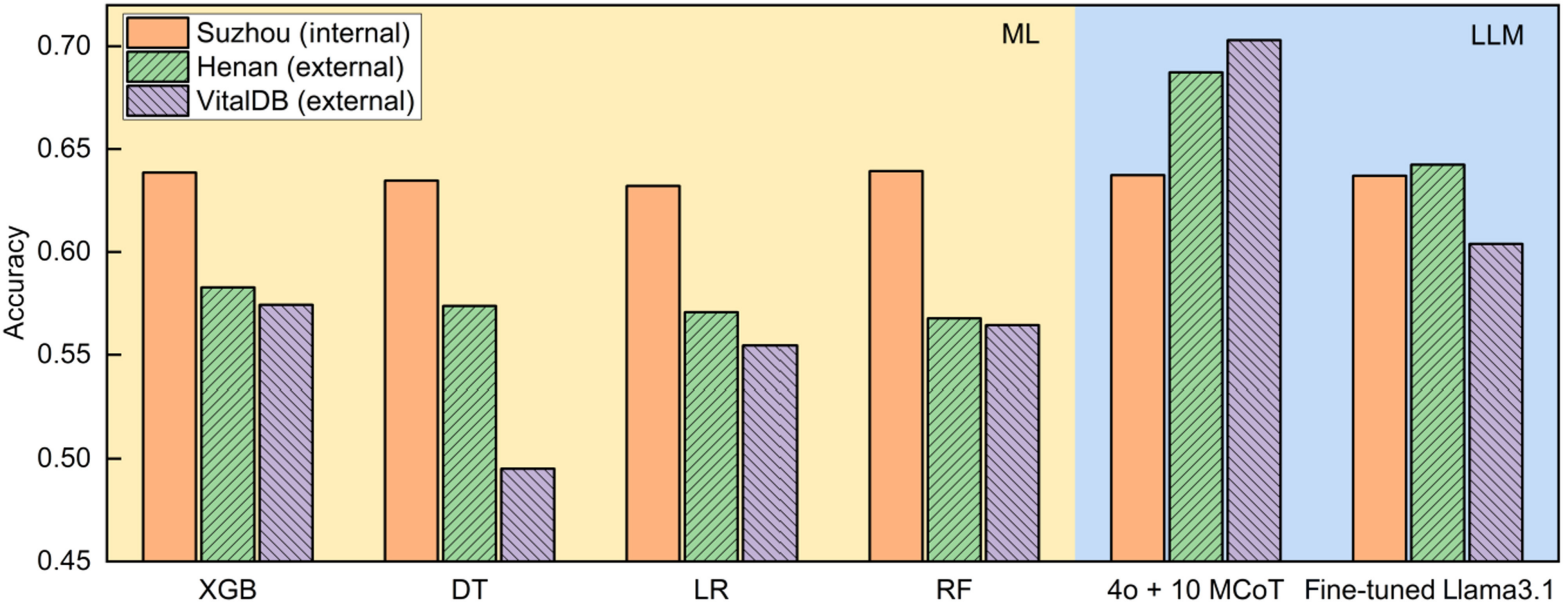
An overview performance of ML models and LLMs. The prediction accuracy of different models across the 3 datasets. The orange bars represent accuracy on the internal Suzhou dataset, the green bars show accuracy on the external Henan dataset, and the purple bars indicate accuracy on the external VitalDB dataset. The light yellow background area encompasses the prediction accuracy of the ML models, while the light blue background area includes the accuracy of GPT-4o under 10-shot MCoT and Llama3.1 after instruction fine-tuning with all available data.

The results indicate that the 4 ML models exhibited similar predictive performances on the Suzhou dataset, with Random Forest achieving the highest performance at 63.93%. However, we noted a significant performance drop between the internal and external datasets: Prediction accuracy for the ML models declined notably on the external datasets. On the internal dataset, GPT-4o based on 10 MCoT and the fine-tuned Llama3.1 achieved accuracies of 63.73% and 63.70%, respectively, both close to the 63.93% achieved by the Random Forest model. On the external dataset, GPT-4o achieved 68.73% and 70.30%, while Llama3.1 reached 64.24% and 60.39%, significantly outperforming the ML models, which had accuracies of 58.27% and 57.43%, respectively. Figure 3 compares the predictive performances of the ML models and the 2 LLM prediction schemes. It shows that while the ML models and LLMs performed similarly on the internal dataset, the LLMs significantly outperformed the ML models on the external datasets.

This reflects ML models’ relatively poor generalization ability on external data, likely due to overfitting during training and differences in feature sets between the internal and external datasets [11]. In contrast, LLMs primarily rely on knowledge from pretraining on large corpora [27]. With the help of prompt engineering and instruction tuning, LLMs can enhance their AKI prediction capabilities and are less impacted by dataset differences, leading to better generalization on external data (Table 4).

**Table 4.** Performance (%) of ML models on the Suzhou, Henan, and VitalDB datasets

	Suzhou (internal)			Henan (external)			VitalDB (external)		
Model	Accuracy	Precision	Recall	Accuracy	Precision	Recall	Accuracy	Precision	Recall
XGBoost	63.86	61.08	81.65	58.27	56.97	66.20	57.43	54.65	92.15
Decision Tree	63.47	65.71	57.51	57.37	55.68	70.60	49.51	50.00	64.71
Logistic Regression	63.21	63.73	61.40	57.07	68.85	25.20	55.45	63.64	27.45
Random Forest	63.93	65.08	79.62	56.77	55.56	66.00	56.44	54.93	76.47

### Explainable interpretability of LLMs and ML models

**Fig. 4.**
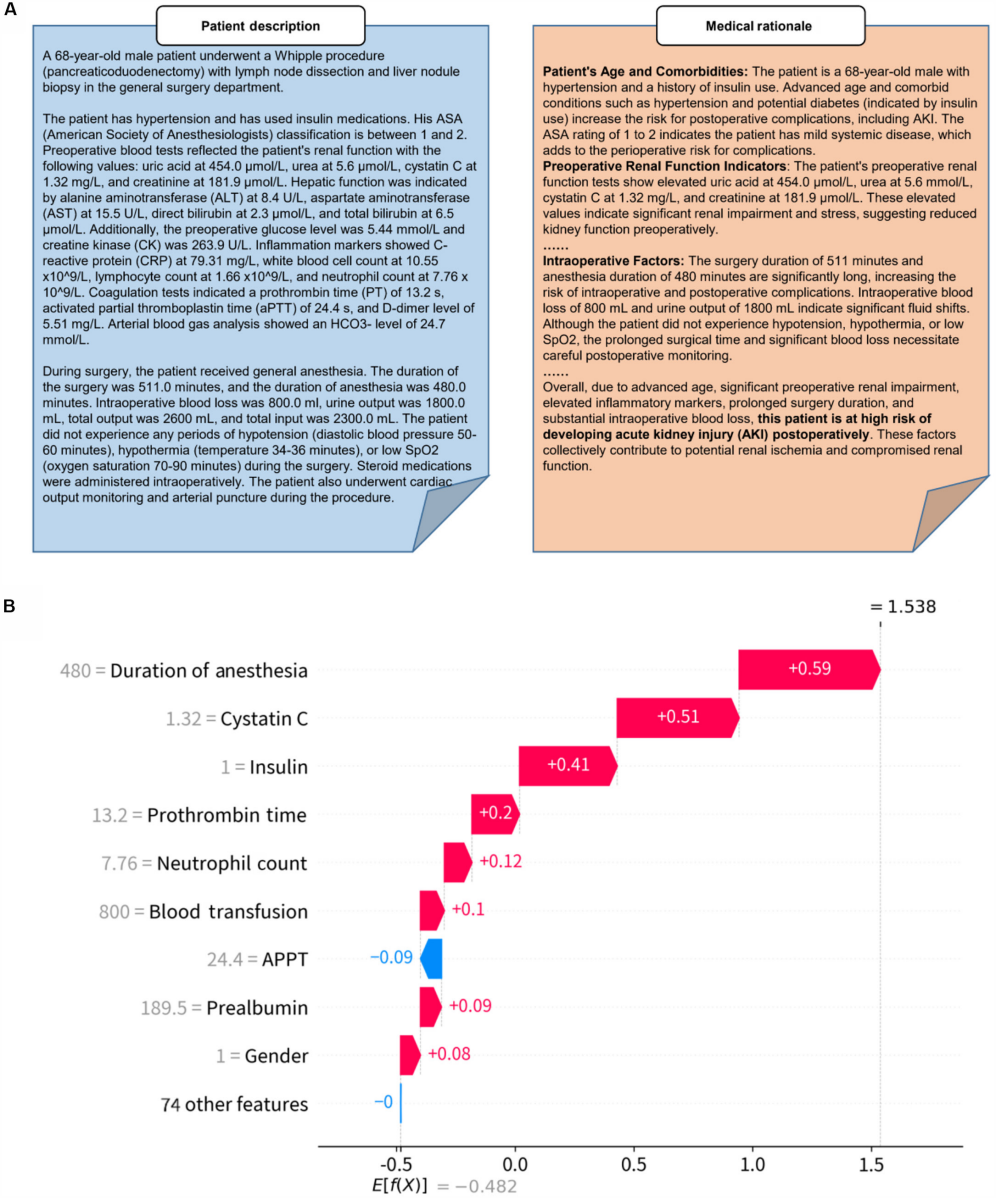
Example of interpretability of LLM and ML model. (A) Example of a medical rationale generated by LLM. The blue section presents textual patient descriptions, including demographic characteristics, medical history, preoperative blood tests, and intraoperative records. This description is input into the LLM, which generates the orange section containing the medical rationale. The medical rationale systematically analyzes the postoperative AKI risk factors in elderly patients and provides a corresponding prediction result. (B) Example of the impact of patient features on prediction results. The impact of patient features on prediction results generated by SHAP, based on the XGBoost model. The patient’s prolonged anesthesia duration was associated with an increased risk of AKI, likely due to the indirect effects of extended anesthesia on other factors such as blood pressure instability or renal perfusion. Elevated levels of cystatin C, a marker for renal function, suggested potential preexisting kidney dysfunction, indicating that the patient may have already had some renal impairment prior to surgery, thus increasing the likelihood of developing AKI.

Figure 4A presents an example of how LLMs generate interpretable medical rationales and prediction results based on patient descriptions. The LLMs gradually analyze AKI-related risk factors, organizing them into readable medical risk points for physicians to reference in further decisions. Traditional ML models typically use the SHapley Additive exPlanations (SHAP) [28] algorithm for feature importance analysis [23]. Based on Shapley values from game theory, SHAP assigns a contribution value to each feature, indicating its importance in the prediction outcome. The key features identified by SHAP often require physicians to use their expertise to interpret why these features impact postoperative AKI patient outcomes.

Table S3 presents the format of the patient information obtained through ML, displayed as structured tabular data that include the values corresponding to the features used in the training. Figure 4B illustrates the impact of patient features on prediction results from the XGBoost model, utilizing the SHAP algorithm. This method is commonly used for interpretability analysis in disease prediction tasks that employ ML models. Compared to the readable text generated by LLMs, the images produced by the SHAP algorithm can be challenging for individuals without medical expertise to interpret, requiring doctors to explain them using their professional knowledge.

In contrast, LLMs offer a more intuitive explanation of predictions. By leveraging the model’s internal medical knowledge, LLMs analyze AKI-related factors—such as demographic characteristics, preoperative kidney function indicators, and intraoperative factors—and then summarize the medical principles and outcomes in a coherent text. This provides physicians with an easily readable format, aiding in the quicker development of appropriate medical interventions. Furthermore, the intuitive medical rationale generated by LLMs can also help non-experts, such as patients and their families, better understand the patient’s AKI risk factors and thus cooperate more effectively with the physician’s intervention strategies.

## Discussion

In this study, we developed 2 types of models—based on commercial LLMs and open-source LLMs—for predicting postoperative AKI outcomes in elderly surgical patients. By incorporating preoperative and intraoperative information, LLMs can analyze features potentially leading to postoperative AKI, generate medical rationales, and predict the likelihood of AKI occurrence. We validated the proposed approach on 2 commercial LLMs, GPT-4o and GPT-4o mini, as well as 2 open-source LLMs, Qwen2-7B-Instruct and Llama3.1-8B-Instruct. Compared to direct predictions from the original models, GPT-4o and GPT-4o mini using MCoT and instruction fine-tuned Qwen2-7B-Instruct and Llama3.1-8B-Instruct improved prediction accuracy for postoperative AKI.

Predicting AKI in a broad surgical population is a challenging task, which explains why the prediction accuracy in this study is below 65%. However, the area under the curve (AUC) value of the receiver operating characteristic (ROC) curve of the ML model reached 74.90% (Table S2), which is comparable to the 74.70% AUC for predicting AKI within 7 d reported in a similar study [29]. Thus, we believe that the model demonstrates sufficient effectiveness, and further research into its generalizability and interpretability is needed.

We observed that commercial and open-source LLMs exhibited high Recall but relatively low Precision in predicting AKI without example-based prompting or fine-tuning. In other words, while most AKI cases were identified, many patients predicted to develop postoperative AKI did not actually experience it. This suggests that without example support, the models tend to make aggressive predictions, forecasting that most patients are at risk of developing postoperative AKI. This phenomenon may indicate a bias in the LLMs’ pretraining data, leading them to associate surgical patients with postoperative AKI outcomes. After adding examples for prompt-based instruction and fine-tuning, the models achieved higher overall prediction accuracy and balanced Recall and Precision. Specifically, Recall decreased slightly while Precision improved, indicating that the model adjusted its threshold for identifying AKI patients. Instead of broadly predicting AKI for most patients, the model conducted a more refined analysis based on the provided examples, resulting in a more balanced prediction.

In many studies using ML models to predict patient conditions across multiple centers, the performance of models trained on single-center data often deteriorates significantly when tested on external datasets from other centers [13,15], wildly when feature distributions vary greatly. This drop in performance is primarily caused by overfitting to single-center data and feature differences between internal and external datasets. To address this issue, numerous studies have explored ways to ensure that models continue to perform well on new datasets with differing characteristics [30].

We observed that LLMs primarily rely on pretraining data during prediction, enabling them to exhibit more robust generalization capabilities across diverse datasets. We evaluated the performance of LLMs under our predictive framework on the internal dataset Suzhou and the external datasets Henan and VitalDB, comparing them with commonly used ML models in AKI prediction across both internal and external datasets. The results showed a significant performance gap for traditional ML models between the internal and external datasets. At the same time, LLMs demonstrated better generalization, maintaining robust predictive performance on unseen external datasets. This suggests that LLMs draw heavily from their rich pretraining data, and by leveraging prompt engineering and instruction fine-tuning, their adaptability to specific tasks can be further enhanced. Therefore, in scenarios involving multi-center medical data with feature discrepancies, LLM-based approaches may better mitigate the impact of data variability on prediction outcomes.

Moreover, given LLMs’ powerful text generation capabilities, we recognized their potential to offer improved interpretability in medical prediction tasks compared to traditional ML approaches [31]. Specifically, LLMs can automatically generate human-readable explanatory texts for physicians, enhancing the transparency of predictions. By enabling the LLMs to sequentially generate medical rationale before making predictions, we successfully obtained explanations for the model’s predictive outputs. Current interpretability methods commonly used in AKI prediction, such as the SHAP algorithm, may reveal feature importance correlations that may lack true causal relationships in clinical practice [32]. In contrast, when predicting AKI, LLMs rely on the medical knowledge embedded in their pretraining data, including textbooks and guidelines, making the generated explanations potentially more meaningful in clinical analysis.

In medical settings, acquiring labeled data can be challenging. Thus, achieving good predictive performance with a small amount of labeled data is practically significant. In the commercial LLM prediction task, we employed only 10 labeled cases as prompts and achieved prediction results comparable to those of ML models across the entire dataset. Hence, LLMs represent a viable alternative for making accurate predictions for diseases where large amounts of labeled data are difficult to obtain.

This study has some limitations. Currently, the LLM-based prediction model directly classifies patients as either “high risk” or “low risk,” rather than outputting a probability value like traditional ML models. In future work, we will explore the performance differences between these 2 output approaches in medical applications. Besides, content generated by LLMs may exhibit hallucination issues [33], such as producing information inconsistent with real-world facts. Many studies are investigating ways to mitigate this phenomenon [34]. In our future work, we plan to incorporate retrieval-augmented generation (RAG) [35] techniques to provide the LLMs with up-to-date medical knowledge on AKI without altering the model parameters. This approach aims to reduce potential factual inaccuracies and explore whether it can further improve prediction accuracy. Additionally, recent studies suggest that agent-based approaches combining multiple LLMs may outperform individual models in disease prediction [36]. For example, by introducing an additional LLM as a learning module, it can receive the prediction results and actual postoperative outcomes, analyze the discrepancies between them, and use the analysis results as a prompt input to the prediction module in this study, enabling the model to continuously learn and improve the credibility and effectiveness of subsequent predictions. We will further investigate whether integrating additional LLMs can enhance the prediction of postoperative AKI in elderly patients.

Recently, LLMs are primarily applied in the medical domain for tasks such as medical text extraction and question-answering related to medical information [37,38]. In this study, we utilized LLMs to predict postoperative AKI outcomes in elderly patients based on EHR data. Experimental results demonstrated favorable predictive performance on both internal and external datasets, showcasing the potential of LLMs in addressing the challenges of complex, multifactorial disease prediction. We hope that this research will inspire further exploration of LLMs in complex disease forecasting.

## Methods

### Data sources

This study utilized 2 Chinese hospital datasets from the First Affiliated Hospital of Soochow University and Henan Provincial People’s Hospital, as well as a publicly available dataset, VitalDB, from South Korea. The Suzhou and Henan datasets included patients aged 65 years and older who underwent anesthesia and surgery between January 2013 and December 2023. The Suzhou dataset comprised 772 postoperative AKI patients and 722 randomly selected non-AKI patients, while the Henan dataset included 500 postoperative AKI patients and 504 randomly selected non-AKI patients. The Suzhou dataset and Henan dataset were approved by the Bioethics Committee of the First Affiliated Hospital of Soochow University (Ethical Research Batch No. 094). In VitalDB, patients aged 65 and older who underwent surgery were selected, yielding 51 postoperative AKI patients and 50 randomly selected non-AKI patients. Postoperative AKI was defined based on the KDIGO criteria [39], using creatinine changes within 7 d post-surgery. Ultimately, the Suzhou dataset was used as the internal dataset, while the Henan and VitalDB datasets served as external validation.

### Data preprocessing

In the Suzhou dataset and Henan dataset, the raw data were provided in tabular format, with time-related information such as surgery and hypotension duration automatically calculated during data extraction. For VitalDB, the raw data included 2 tables and a series of waveform files capturing intraoperative physiological information recorded by medical devices. From the VitalDB table, timestamps were used to calculate surgery and anesthesia durations, and drug administration features were transformed into binary features indicating whether specific medications were used. Intraoperative physiological features such as hypotension and hypothermia duration were computed by extracting information from the waveform files. Given the differences in available features between VitalDB and the other 2 datasets, features absent in VitalDB were filled with zeros when testing ML models that could not handle missing values, such as logistic regression. Laboratory results not provided in VitalDB were imputed using mean values from the internal dataset.

### LLM MCoT dataset generation

Since LLMs cannot directly process structured tabular data, we utilized Python scripts to convert the preprocessed data into textual descriptions, including patient information and postoperative AKI outcomes, allowing LLMs to fully leverage their text processing capabilities. To avoid interference from multilingual factors, all converted text was standardized to English. From the Suzhou dataset, 544 cases were randomly selected. Based on Prompt 1 (Fig. S1), the patient descriptions and AKI outcomes were input into GPT-4o, where medical rationales were generated under physician supervision. These medical rationales, along with the corresponding patient descriptions and AKI outcomes, were compiled into the MCoT dataset.

### Prompt engineering and testing with commercial LLMs

Initially, based on Prompt 2 (Fig. S1), patient descriptions were input into GPT-4o and GPT-4o mini to predict postoperative AKI outcomes. Zero-shot and few-shot methods were tested on 3 datasets. Subsequently, in the MCoT sample, physicians selected 10 representative cases as MCoT shots for future reference. Based on Prompt 3 (Fig. S1), patient descriptions were again input into GPT-4o and GPT-4o mini to request both medical rationale and AKI outcomes. The zero-shot MCoT and few-shot MCoT methods were tested on 3 datasets. In this study, the few-shot prompt is first tokenized and then transformed into vector representations through embedding layers. LLMs based on the Transformer architecture process these vector representations using a self-attention mechanism, which dynamically adjusts the correlations between different tokens within the context. This mechanism directs the model’s focus to input segments that are relevant to the specific task. Through this process, LLMs can identify and extract task-specific patterns and structures from the few-shot prompt. Leveraging these identified patterns, LLMs perform reasoning to generate the corresponding predictive text. Overall, this approach enables LLMs to effectively utilize the few-shot prompt for accurate and contextually appropriate text generation. Testing was conducted using the API (application programming interface) provided by commercial LLM services, with a max_tokens of 4,096, temperature of 0.7, top_p of 1.0, and *n* of 1. To ensure result stability, the final results were averaged over 5 runs.

### Instruction fine-tuning and testing with open-source LLMs

Using the instructions and format provided in Fig. S2, we created fine-tuning training data by pairing patient descriptions as inputs with medical rationales and AKI outcomes as outputs from the generated MCoT samples. In this study, we employed the low-rank adaptation (LoRA) method for instruction tuning. LoRA is a lightweight fine-tuning technique that introduces trainable low-rank matrices into the original weight matrices, adjusting only the parameters of these low-rank matrices. This approach significantly reduces the computational and storage overhead required for fine-tuning LLMs. Considering that the objective of this study is to validate the effectiveness of the proposed method, we did not employ a variety of instructions during the fine-tuning process. Instead, we utilized specific instructions designed in Fig. S2 for training, facilitating the use of the same instructions in subsequent testing. LoRA fine-tuning was conducted using llama-factory [40], with a learning rate of 2 × 10^−5^, a cosine learning rate scheduler, 10 training epochs, and 20 warmup steps. In our experiments, we applied LoRA to fine-tune the weight matrices of the query, key, value, up, down, gate, and output. After fine-tuning, the model was tested on 1,000 nontuned cases from the internal dataset and on 2 external datasets by inputting instructions and patient descriptions to generate medical rationales and AKI outcomes. Testing was performed via a local API with do_sample set to true, max_tokens of 4,000, temperature of 0.7, top_p of 1.0, and *n* of 1. To ensure result stability, the final results were averaged over 5 runs. The deployment and fine-tuning of the LLMs were run on a single NVIDIA RTX 4090 GPU, with 24 GB of RAM.

### ML model training and testing

We selected ML models commonly used for AKI prediction, including XGBoost, Decision Tree, Logistic Regression, and Random Forest, for testing. All models were executed using Python 3.11.9, with the XGBoost model implemented via the Python package xgboost and the Decision Tree, Logistic Regression, and Random Forest models implemented using modules from the sklearn package. In the ML process, we converted categorical variables into numerical values and trained the model alongside the remaining numerical variables. To ensure consistency with the information used in the LLM framework and maintain the model’s interpretability, we retained all the variables in the ML model and did not apply common dimensionality reduction techniques such as principal components analysis (PCA). The hyperparameters of the ML model were selected through manual design to prevent overfitting, thereby ensuring the model’s generalizability on external datasets. In the internal dataset, we employed 5-fold cross-validation for testing. For external dataset validation, the models were first trained on the internal dataset, resulting in predictive models. The threshold that achieved the highest accuracy in internal testing was then selected. Using the trained prediction models and the selected thresholds, we made predictions on the external datasets to obtain the final results.

### Performance evaluation

In our dataset, the AKI labels are binary, indicating whether patients developed AKI within 7 d after surgery. Consequently, we have formulated our prediction task as a binary classification problem to assess whether a patient is at a “high risk” or “low risk” of developing postoperative AKI. As such, all evaluation metrics discussed in this paper are based on binary classification results. Since the datasets were balanced, we adopted Accuracy, Precision, and Recall to evaluate the models’ predictive performance. Accuracy reflects the overall performance of the model, Precision indicates the accuracy when predicting positive outcomes, and Recall demonstrates the model’s ability to identify positive samples. These 3 metrics allow us to assess the model’s overall performance. In this study, we primarily focus on the model’s Accuracy.

## Data Availability

The Suzhou and Henan datasets cannot be made publicly available because of the potential risk to patient privacy. However, relevant data are available upon reasonable request. The VitalDB dataset is available from https://vitaldb.net/. The original open-source models Qwen2-7B-Instruct and Llama3.1-8B-Instruct can be downloaded from https://huggingface.co/Qwen/Qwen2-7B-Instruct and https://huggingface.co/meta-llama/Meta-Llama-3.1-8B-Instruct, respectively. The code of data preprocessing, machine learning, 2 MCoT samples, and instruction fine-tuning scripts is available in our GitHub repository (https://github.com/warriorod/LFPAP).

## References

[1] Ronco C, Bellomo R, Kellum JA. Acute kidney injury. Lancet. 2019;394(10212):1949–1964. 31777389 10.1016/S0140-6736(19)32563-2

[2] Joosten A, Lucidi V, Lckx B, Van Obbergh L, Germanova D, Berna A, Alexander B, Desebbe O, Carrier F-M, Cherqui D, et al. Intraoperative hypotension during liver transplant surgery is associated with postoperative acute kidney injury: A historical cohort study. BMC Anesthesiol. 2021;21(1):12. 33430770 10.1186/s12871-020-01228-y PMC7798188

[3] Zhang Y, Jiang L, Wang B, Xi X. Epidemiological characteristics of and risk factors for patients with postoperative acute kidney injury: A multicenter prospective study in 30 Chinese intensive care units. Int Urol Nephrol. 2018;50(7):1319–1328. 29480442 10.1007/s11255-018-1828-7

[4] Stille K, Kribben A, Herget-Rosenthal S. Incidence, severity, risk factors and outcomes of acute kidney injury in older adults: Systematic review and meta-analysis. J Nephrol. 2022;35(9):2237–2250. 35932418 10.1007/s40620-022-01381-2

[5] Privratsky JR, Fuller M, Raghunathan K, Ohnuma T, Bartz RR, Schroeder R, Price TM, Martinez MR, Sigurdsson MI, Mathis MR, et al. Postoperative acute kidney injury by age and sex: A retrospective cohort association study. Anesthesiology. 2023;138(2):184–194. 36512724 10.1097/ALN.0000000000004436 PMC10439699

[6] Meersch M, Schmidt C, Hoffmeier A, Von Aken H, Wempe C, Gerss J, Zarbock A. Prevention of cardiac surgery-associated AKI by implementing the KDIGO guidelines in high risk patients identified by biomarkers: The PrevAKI randomized controlled trial. Intensive Care Med. 2017;43(11):1551–1561. 28110412 10.1007/s00134-016-4670-3 PMC5633630

[7] Qu C, Gao L, Yu X-Q, Wei M, Fang G-Q, He J, Cao L-X, Ke L, Tong Z-H, Li W-Q. Machine learning models of acute kidney injury prediction in acute pancreatitis patients. Gastroenterol Res Pract. 2020;2020:3431290. 33061958 10.1155/2020/3431290 PMC7542489

[8] Linardatos P, Papastefanopoulos V, Kotsiantis S. Explainable AI: A review of machine learning interpretability methods. Entropy. 2020;23(1):18. 33375658 10.3390/e23010018 PMC7824368

[9] Williams MM, Kemp BR, Ferraro KF. Diverse aging and health inequality by race and ethnicity. Innov Aging. 2017;1(1):igx002. 29795805 10.1093/geroni/igx002 PMC5954610

[10] Yuan L, Yu B, Gao L, Du M, Lv Y, Liu X, Sun J. Decomposition analysis of health inequalities between the urban and rural oldest-old populations in China: Evidence from a national survey. SSM Population Health. 2023;21:101325. 36618546 10.1016/j.ssmph.2022.101325 PMC9816804

[11] Cabitza F, Campagner A, Soares F, de Guadiana-Romualdo LG, Challa F, Sulejmani A, Seghezzi M, Carobene A. The importance of being external. Methodological insights for the external validation of machine learning models in medicine. Comput Methods Prog Biomed. 2021;208: Article 106288. 10.1016/j.cmpb.2021.106288 34352688

[12] Dutta S, McEvoy DS, Dunham LN, Pharm RS, Rubins D, McMahon GM, Samal L. External validation of a commercial acute kidney injury predictive model. NEJM AI. 2024;1(3):AIoa2300099.

[13] Ohnuma T, Uchino S, Toki N, Tekada K, Namba Y, Katayama S, Kawarazaki H, Yasuda H, Izawa J, Uji M, et al. External validation for acute kidney injury severity scores: A multicenter retrospective study in 14 Japanese ICUs. Am J Nephrol. 2015;42(1):57–64. 26337793 10.1159/000439118

[14] Patidar KR, Xu C, Shamseddeen H, Cheng Y-W, Ghabril MS, Mukthinuthalapati VVPK, Fricker ZP, Akinyeye S, Nephew LD, Desai AP, et al. Development and validation of a model to predict acute kidney injury in hospitalized patients with cirrhosis. Clin Transl Gastroenterol. 2019;10(9): Article e00075. 31478958 10.14309/ctg.0000000000000075 PMC6775340

[15] Li Q, Lv H, Chen Y, Shen J, Shi J, Zhou C. Development and validation of a machine learning predictive model for cardiac surgery-associated acute kidney injury. J Clin Med. 2023;12(3):1166. 36769813 10.3390/jcm12031166 PMC9917969

[16] Liu F, Zhu T, Wu X, Yang B, You C, Wang C, Lu L, Liu Z, Zheng Y, Sun X, et al. A medical multimodal large language model for future pandemics. NPJ Digit Med. 2023;6(1):226. 38042919 10.1038/s41746-023-00952-2 PMC10693607

[17] Wu H, Boulenger P, Faure A, Cespedes B, Boukil F, Morel N, Chen Z, Bosselut A. EPFL-MAKE at “discharge me!”: An LLM system for automatically generating discharge summaries of clinical electronic health record. Paper presented at: Proceedings of the 23rd Workshop on Biomedical Natural Language Processing; August 2024; Bangkok, Thailand.

[18] Banu GS, Joita D, Priescu I, The transformative role of large language models in medicine. Paper presented at: Advances in Digital Health and Medical Bioengineering (IFMBE Proceedings); 2023 Nov 9–10; Bucharest, Romania.

[19] Scanlon M, Breitinger F, Hargreaves C, Hilgert J-N, Sheppard J. ChatGPT for digital forensic investigation: The good, the bad, and the unknown. Forensic Sci Int Dig Invest. 2023;46:301609.

[20] Gou J, Yu B, Maybank SJ, Tao D. Knowledge distillation: A survey. Int J Comput Vis. 2021;129(6):1789–1819.

[21] Lee HC, Park Y, Yoon SB, Yang SM, Park D, Jung CW. VitalDB, a high-fidelity multi-parameter vital signs database in surgical patients. Sci Data. 2022;9(1):279. 35676300 10.1038/s41597-022-01411-5 PMC9178032

[22] Wei J, Wang Z, Schuurmans D, Bosma M, Ichter B, Xia F, Chi EH, Le QV, Zhou D, et al. Chain-of-thought prompting elicits reasoning in large language models. Adv Neural Inf Proces Syst. 2022;35:24824–24837.

[23] Hu J, Xu J, Li M, Jiang Z, Mao J, Feng L, Miao K, Li H, Chen J, Bai Z, et al. Identification and validation of an explainable prediction model of acute kidney injury with prognostic implications in critically ill children: A prospective multicenter cohort study. EClinicalMedicine. 2024;68: Article 102409. 38273888 10.1016/j.eclinm.2023.102409 PMC10809096

[24] Kate RJ, Perez RM, Mazumdar D, Pasupathy KS, Nilakantan V. Prediction and detection models for acute kidney injury in hospitalized older adults. BMC Med Inform Decis Mak. 2016;16:39. 27025458 10.1186/s12911-016-0277-4 PMC4812614

[25] Xin W, Yi W, Liu H, Haixia L, Dongdong L, Ma Y, Li G. Early prediction of acute kidney injury after liver transplantation by scoring system and decision tree. Ren Fail. 2021;43(1):1137–1145. 34261422 10.1080/0886022X.2021.1945462 PMC8281092

[26] Zhang Z, Ho KM, Hong Y. Machine learning for the prediction of volume responsiveness in patients with oliguric acute kidney injury in critical care. Crit Care. 2019;23(1):112. 30961662 10.1186/s13054-019-2411-z PMC6454725

[27] Guimarães N, Campos R, Jorge A. Pre-trained language models: What do they know? WIREs Data Min Knowl Disc. 2023;14(1):e1518.

[28] Scott M, Su-In L. A unified approach to interpreting model predictions. Adv Neural Inf Proces Syst. 2017;30:4765–4774.

[29] Cheng P, Waitman LR, Hu Y, Liu M. Predicting inpatient acute kidney injury over different time horizons: How early and accurate? Paper presented at: AMIA Annual Symposium Proceedings; 2017 Nov 4–8; Washington, DC, USA. PMC5977670 29854121

[30] Khoee AG, Yu Y, Feldt R. Domain generalization through meta-learning: A survey. Artif Intell Rev. 2024;57(10):285.

[31] Kwon T, Ong KT, Kang D, Moon S, Lee JR, Hwang D, Sohn B, Sim Y, Lee D, Yeo J. Large language models are clinical reasoners: Reasoning-aware diagnosis framework with prompt-generated rationales. Proc AAAI Conf Artif Intell. 38(16):18417–18425.

[32] Slack D, Hilgard S, Jia E, Singh S, Lakkaraju H. Fooling LIME and SHAP. Paper presented at: Proceedings of the AAAI/ACM Conference on AI, Ethics, and Society; 2020 Feb 7–8; New York, NY, USA.

[33] McGowan A, Gui Y, Dobbs M, Shuster S, Cotter M, Selloni A, Goodman M, Srivastava A, Cecchi GA, Corcoran CM. ChatGPT and bard exhibit spontaneous citation fabrication during psychiatry literature search. Psychiatry Res. 2023;326: Article 115334. 37499282 10.1016/j.psychres.2023.115334 PMC10424704

[34] Lin Z, Guan S, Zhang W, Zhang H, Li Y, Zhang H. Towards trustworthy LLMs: A review on debiasing and dehallucinating in large language models. Artif Intell Rev. 2024;57(9):243.

[35] Lewis P, Perez E, Piktus A, Petroni F, Karpukhin V, Goyal N, Kuttler H, Lewis M, Yih W-T, Rocktaschel T, et al. Retrieval-augmented generation for knowledge-intensive NLP tasks. Adv Neural Inf Proces Syst. 2020;33:9459–9474.

[36] Cui H, Shen Z, Zhang J, Shao H, Qin L, Ho JC, Yang C. LLMs-based few-shot disease predictions using EHR: A novel approach combining predictive agent reasoning and critical agent instruction. arXiv. 2024. 10.48550/arXiv.2403.15464. PMC12099430 40417470

[37] Yeo YH, Samaan JS, Ng WH, Ting P-S, Trivedi H, Vipani A, Ayoub W, Yang JD, Liran O, Spiegel B, et al. Assessing the performance of ChatGPT in answering questions regarding cirrhosis and hepatocellular carcinoma. Clin Mol Hepatol. 2023;29(3):721–732. 36946005 10.3350/cmh.2023.0089 PMC10366809

[38] Gu Z, He X, Yu P, Jia W, Yang X, Peng G, Hu P, Chen S, Chen H, Lin Y. Automatic quantitative stroke severity assessment based on Chinese clinical named entity recognition with domain-adaptive pre-trained large language model. Artif Intell Med. 2024;150: Article 102822. 38553162 10.1016/j.artmed.2024.102822

[39] Prowle JR, Forni LG, Bell M, Chew MS, Edwards M, Grams ME, Grocott MPW, Liu KD, Mcllroy D, Murray PT, et al. Postoperative acute kidney injury in adult non-cardiac surgery: Joint consensus report of the acute disease quality initiative and perioperative quality initiative. Nat Rev Nephrol. 2021;17(9):605–618. 33976395 10.1038/s41581-021-00418-2 PMC8367817

[40] Zheng Y, Zhang R, Zhang J, Ye Y, Luo Z. Llamafactory: Unified efficient fine-tuning of 100+ language models. arXiv. 2024. 10.48550/arXiv.2403.13372.

