# Supplementary material for: Leveraging Large Language Models for Predicting Postoperative Acute Kidney Injury in Elderly Patients
Source: BME Front. 2025 Mar 11;6:0111. doi: 10.34133/bmef.0111 (PMC11896637; doi:10.34133/bmef.0111)
Supplement: Supplementary 1 — Figs. S1 and S2 Tables S1 to S3 [file bmef.0111.f1.docx]

Supplementary Materials for

**Leveraging Large Language Models for Predicting Postoperative Acute Kidney Injury in Elderly Patients**

*Hanfei Zhu, Ruojiang Wang *, Jiajie Qian, Yuhao Wu, Zhuqing Jin, Xishen Shan, Fuhai Ji, Zixuan Yuan and Tingrui Pan **

*Corresponding authors. Ruojiang Wang (ruojiang@ustc.edu.cn) and Tingrui Pan (tingrui@ustc.edu.cn)

**This PDF file includes:**

Supplementary Figures 1 to 2

Supplementary Tables 1 to 3


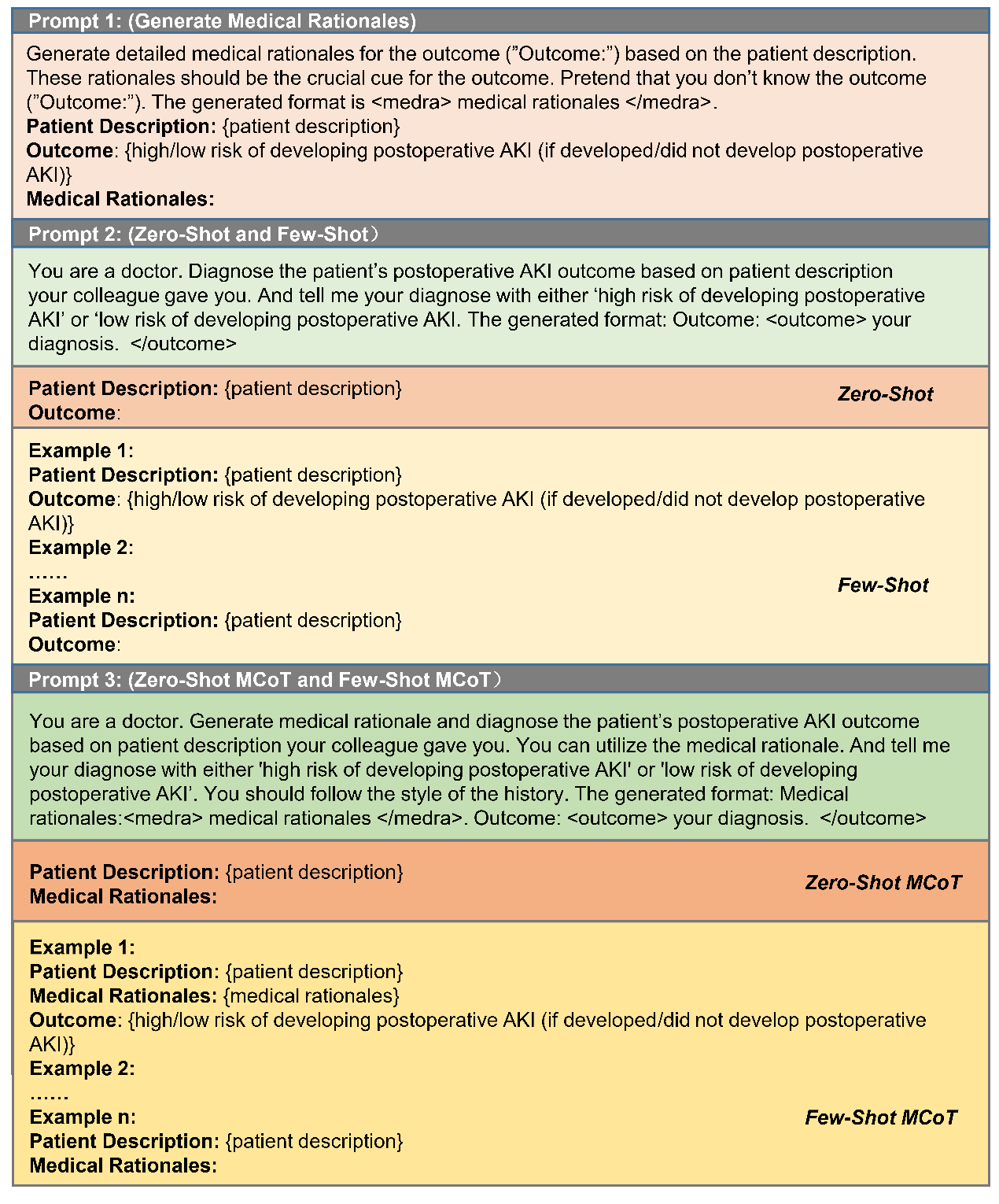
**Supplementary Figure 1.** Prompts for commercial LLMs.


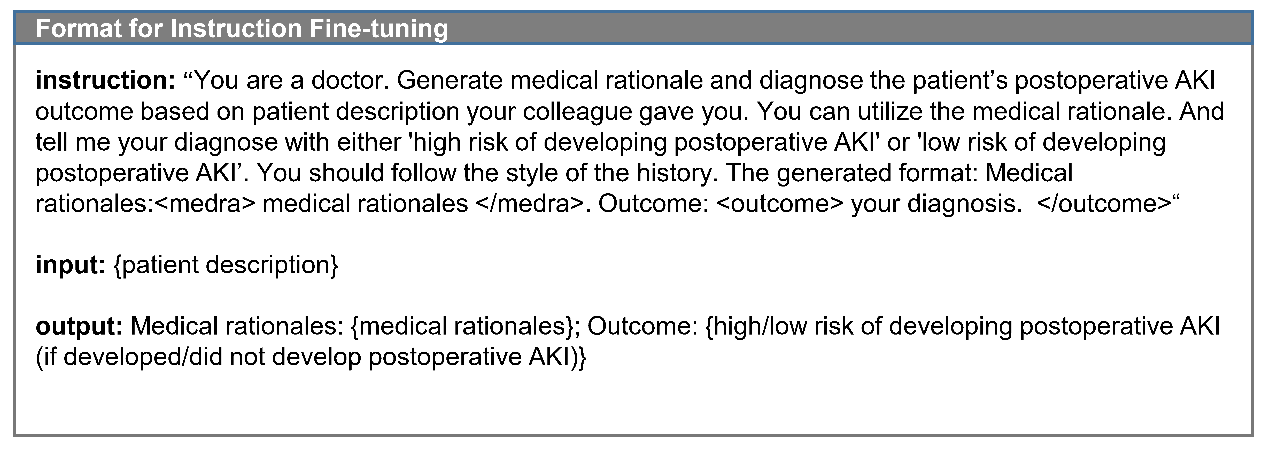
**Supplementary Figure 2.** Format for instruction fine-tuning open-source LLMs.

**Supplementary Table 1.** All characteristics of the Suzhou, Henan, and VitalDB datasets.

| Dataset | Suzhou (Internal) | Henan (External) | VitalDB (External) |
| --- | --- | --- | --- |
| Gender (M:F) | 869:675 | 555:449 | 72:29 |
| Age (Mean, SD) | 71.76 (5.92) | 73.16 (6.79) | 71.93 (5.54) |
| Use of Insulin (Number, %) | 312 (20.21) | 291 (21.81) | / |
| Use of ACEI/ARB (Number, %) | 273 (17.68) | 43 (4.28) | / |
| Hypertension (Number, %) | 725 (46.96) | 248 (24.73) | 48 (47.52) |
| Diabetes (Number, %) | 210 (13.60) | 213 (21.22) | 15 (14.85) |
| Coronary Heart Disease (Number, %) | 95 (6.15) | 148 (14.74) | / |
| Cerebral Infarction (Number, %) | 118 (7.64) | 153 (15.24) | / |
| Kidney Disease (Number, %) | 58 (3.76) | 12 (1.20) | / |
| Thrombosis (Number, %) | 6 (0.39) | 16 (1.59) | / |
| Emphysema (Number, %) | 6 (0.39) | 17 (1.69) | / |
| Bronchitis (Number, %) | 3 (0.19) | 8 (0.80) | / |
| Heart Failure (Number, %) | 3 (0.19) | 9 (0.90) | / |
| Glucose *mmol/L* (Mean, SD) | 5.84 (1.97) | 5.95 (2.28) | 7.30 (2.92) |
| Uric Acid *μmol/L* (Mean, SD) | 311.29 (106.17) | 299.89 (98.46) | / |
| Blood Urea Nitrogen *mmol/L* (Mean, SD) | 6.07 (2.60) | 6.49 (2.45) | 1.08 (0.78) |
| Creatinine *mmol/L* (Mean, SD) | 70.25 (39.81) | 70.24 (32.21) | 101.18 (117.98) |
| Alanine Aminotransferase *U/L* (Mean, SD) | 21.49 (28.15) | 29.97 (55.98) | 51.62 (243.34) |
| Aspartate Aminotransferase *U/L* (Mean, SD) | 25.05 (32.29) | 30.95 (51.47) | 32.36 (62.59) |
| Direct Bilirubin *μmol/L* (Mean, SD) | 6.16 (9.10) | 8.48 (26.16) | / |
| Total Bilirubin *μmol/L* (Mean, SD) | 16.18 (13.12) | 17.43 (34.04) | 15.43 (18.93) |
| Albumin *g/L* (Mean, SD) | 38.88 (4.97) | 37.71 (4.50) | 36.88 (5.45) |
| Potassium *mmol/L* (Mean, SD) | 3.93 (0.43) | 4.11 (0.44) | 5.45 (13.46) |
| Sodium *mmol/L* (Mean, SD) | 140.82 (2.80) | 140.43 (3.30) | 139.34 (3.29) |
| Calcium *mmol/L* (Mean, SD) | 2.23 (0.18) | 2.20 (0.16) | 1.09 (0.10) |
| Lactate Dehydrogenase *U/L* (Mean, SD) | 193.52 (65.77) | 219.90 (166.51) | / |
| Cystatin C *mg/L* (Mean, SD) | 1.16 (0.64) | 1.17 (0.41) | / |
| White Blood Cell Count *x10^9/L* (Mean, SD) | 7.03 (3.05) | 6.97 (3.94) | 7.80 (3.75) |
| Lymphocyte Count *x10^9/L* (Mean, SD) | 1.53 (0.76) | 1.68 (3.08) | / |
| Neutrophil Count *x10^9/L* (Mean, SD) | 4.92 (3.03) | 4.67 (2.48) | / |
| Eosinophil Count *x10^9/L* (Mean, SD) | 0.12 (0.13) | 0.14 (0.13) | / |
| Red Blood Cell Count *x10^9/L* (Mean, SD) | 4.20 (0.60) | 4.02 (0.58) | / |
| Hemoglobin (Mean, SD) | 127.05 (19.38) | 121.95 (17.94) | 118.22 (26.06) |
| Platelet Count *x10^9/L* (Mean, SD) | 196.84 (67.72) | 210.11 (79.12) | 215.66 (83.91) |
| Prothrombin Time *sec* (Mean, SD) | 12.22 (1.86) | 12.18 (1.33) | 12.84 (3.79) |
| Activated Partial Thromboplastin Time *sec* (Mean, SD) | 30.51 (13.65) | 31.82 (5.96) | 33.10 (9.49) |
| Type of Surgery (Text) | / | / | / |
| Surgical Department (Text) | / | / | / |
| General Anesthesia (Number, %) | 1431 (92.68) | 792 (78.88) | 99 (98.02) |
| ASA12 (Number, %) | 1283 (83.10) | 673 (67.03) | 69 (68.32) |
| ASA3 (Number, %) | 250 (16.19) | 316 (31.47) | 28 (27.72) |
| Duration of Operation *min* (Mean, SD) | 193.83 (128.57) | 164.21 (116.95) | 193.15 (127.46) |
| Duration of Anesthesia *min* (Mean, SD) | 168.48 (121.63) | 178.46 (120.94) | 261.64 (139.23) |
| Intraoperative Blood Loss *ml* (Mean, SD) | 178.60 (377.15) | 172.60 (318.23) | 919.98 (2618.24) |
| Urine Output *ml* (Mean, SD) | 241.01 (397.03) | 371.00 (440.17) | 228.56 (231.85) |
| Total Output *ml* (Mean, SD) | 395.65 (673.83) | 543.41 (635.44) | 918.84 (2409.56) |
| Blood Transfusion Volume *ml* (Mean, SD) | 139.92 (383.80) | 50.54 (185.51) | 409.90 (1506.06) |
| Total Input *ml* (Mean, SD) | 1206.75 (874.76) | 1224.72 (1220.11) | 2020.50 (2511.80) |
| Duration of Intraoperative Hypotension *min* (Mean, SD) | 13.90 (31.73) | 54.21 (77.98) | 17.23 (34.57) |
| Duration of Intraoperative Hypothermia *min* (Mean, SD) | 21.11 (57.39) | 0.27 (8.52) | 137.88 (117.69) |
| Duration of Intraoperative Low SpO2 *min* (Mean, SD) | 2.48 (11.96) | 0.57 (4.36) | 3.04 (6.87) |
| Duration of Intraoperative Tachycardia *min* (Mean, SD) | 7.98 (29.47) | 5.58 (25.32) | 23.64 (60.62) |
| Use of Rocuronium Bromide (Number, %) | 433 (28.04) | 2 (0.20) | 95 (94.06) |
| Use of Atracurium (Number, %) | 1070 (69.30) | 419 (41.73) | / |
| Use of Ketamine (Number, %) | 69 (4.47) | 2 (0.20) | / |
| Use of Oxycodone (Number, %) | 63 (4.08) | 0 (0.00) | / |
| Use of Epinephrine (Number, %) | 26 (1.68) | 0 (0.00) | 8 (7.92) |
| Use of Norepinephrine (Number, %) | 122 (7.25) | 46 (4.58) | / |
| Use of Dopamine (Number, %) | 6 (0.39) | 15 (1.49) | / |
| Use of Dobutamine (Number, %) | 1 (0.06) | 0 (0.00) | / |
| Use of Ephedrine (Number, %) | 206 (13.34) | 0 (0.00) | 64 (63.37) |
| Use of Atropine (Number, %) | 48 (3.11) | 0 (0.00) | / |
| Use of Phenylephrine (Number, %) | 152 (9.84) | 2 (0.20) | 35 (34.65) |
| Use of Metaraminol (Number, %) | 45 (2.91) | 0 (0.00) | / |
| Use of Esmolol (Number, %) | 29 (1.88) | 3 (0.30) | / |
| Use of Perdipine (Number, %) | 41 (2.66) | 0 (0.00) | / |
| Use of Nitroglycerin (Number, %) | 3 (0.19) | 15 (1.49) | / |
| Use of Hydrocortisone (Number, %) | 1 (0.06) | 0 (0.00) | / |
| Use of Succinyl Hydrocortisone (Number, %) | 1 (0.06) | 0 (0.00) | / |
| Use of Methylprednisolone (Number, %) | 114 (7.38) | 0 (0.00) | / |
| Use of Dexamethasone (Number, %) | 1120 (72.54) | 0 (0.00) | / |
| Use of Mannitol (Number, %) | 6 (0.39) | 5 (0.50) | / |
| Use of Human Albumin (Number, %) | 29 (1.88) | 0 (0.00) | / |
| Use of Red Blood Cells (Number, %) | 187 (12.11) | 81 (8.07) | / |
| Use of Plasma (Number, %) | 55 (3.56) | 83 (8.27) | / |
| Use of Stored Autologous Blood Transfusion (Number, %) | 9 (0.58) | 12 (1.20) | / |
| Use of Cryoprecipitate (Number, %) | 25 (1.62) | 3 (0.30) | / |
| Use of Succinyl Gelatin (Number, %) | 484 (31.35) | 260 (25.90) | / |
| Use of Polygeline (Number, %) | 370 (23.96) | 0 (0.00) | / |
| Use of Hydroxyethyl Starch (Number, %) | 258 (16.71) | 152 (15.14) | / |
| Use of Tourniquet (Number, %) | 23 (1.49) | 2 (0.20) | / |
| Use of Cardiac Output Monitoring (Number, %) | 120 (7.77) | 0 (0.00) | / |
| Use of Anesthesia Depth Monitoring (Number, %) | 193 (12.50) | 2 (0.20) | / |
| Use of Nerve Block Method (Number, %) | 30 (1.94) | 111 (11.06) | / |
| Use of Arterial Puncture (Number, %) | 1013 (65.61) | 177 (17.63) | / |
| Postoperative AKI Outcome (Number, %) | 772 (50.00) | 500 (49.80) | 51 (50.50) |

**Supplementary Table 2.** The Area Under the Curve (AUC) value (%) of the receiver operating characteristic (ROC) curve of machine learning models in Suzhou dataset. These results are the average obtained from 5-fold cross-validation.

| Model | XGBoost | Decision Tree | Logistic Regression | Random Forest |
| --- | --- | --- | --- | --- |
| AUC | 74.63 | 74.07 | 74.26 | 74.90 |

**Supplementary Table 3:** Structured tabular data of patient features (only a subset of features displayed)

| Gender | Age | Hypertension | Diabetes | Blood Urea Nitrogen | Duration of Anesthesia | … |
| --- | --- | --- | --- | --- | --- | --- |
| M | 68 | 1 | 0 | 5.6 | 480 | … |
